# Early life history responses and phenotypic shifts in a rare endemic plant responding to climate change

**DOI:** 10.1093/conphys/coz076

**Published:** 2019-10-31

**Authors:** Daniel E Winkler, Michelle Yu-Chan Lin, José Delgadillo, Kenneth J Chapin, Travis E Huxman

**Affiliations:** 1 Ecology & Evolutionary Biology, 321 Steinhaus Hall, University of California, Irvine, CA, 92697, USA; 2 United States Geological Survey, 2290 S West Resource Boulevard, Southwest Biological Science Center, UT, 84532, USA; 3 Biological Sciences, University of California, Irvine, CA, 92617, USA; 4 Facultad de Ciencias, Universidad Autónoma de Baja California, Ensenada, Baja California, 22800, México; 5 Ecology & Evolutionary Biology, University of Arizona, P.O. Box 210088, Tucson, AZ, 85721, USA

**Keywords:** Baja California, chasmophyte, cushion, endemic, germination, *Heterotheca*, México, outcropping, phenotypic variation, regeneration niche, seedling establishment, Sierra de San Pedro Mártir

## Abstract

Changes in species ranges are anticipated with climate change, where in alpine settings, fragmentation and contraction are likely. This is especially true in high altitude biodiversity hotspots, where warmer growing seasons and increased drought events may negatively impact populations by limiting regeneration. Here, we test for high-altitude species responses to the interactive effects of warming and drought in *Heterotheca brandegeei*, a perennial cushion plant endemic to alpine outcroppings in Sierra de San Pedro Mártir National Park, Baja California, México. We exposed *H. brandegeei* seedlings to experimental warming and drought conditions to document early life history responses and the species ability to tolerate climate change. Drought negatively influenced seedling growth, with overall reductions in above- and belowground biomass. Warming and drought each led to substantial reductions in leaf development. At the same time, individuals maintained high specific leaf area and carbon investment in leaves across treatments, suggesting that existing phenotypic variation within populations may be high enough to withstand climate change. However, warming and drought interacted to negatively influence leaf-level water-use efficiency (WUE). Seedling mortality rates were nearly three times higher in warming and drought treatments, suggesting bleak prospects for *H. brandegeei* populations in future climate conditions. Overall, our results suggest *H. brandegeei* populations may experience substantial declines under future warmer and drier conditions. Some individuals may be able to establish, albeit, as smaller, more stressed plants. These results further suggest that warming alone may not be as consequential to populations as drought will be in this already water-limited system.

## Teaser

We studied how a rare, endemic alpine cushion plant responds to the interactive effects of warming and drought. Overall, we found that both drought and warming negatively influenced the species growth but that existing levels of phenotypic variation may be enough to at least temporarily buffer populations.

## Introduction

High-elevation systems are predicted to be especially sensitive to climate change (Nagy and Grabherr, 2009; Stocker *et al.*, 2013). These include alpine ecosystems that exist at or near the terminus of mountains, suggesting that upward range shifts in response to climate warming will be limited by habitat availability (Canone *et al.*, 2007; Rixen and Wipf, 2017). Here, a majority of plant species are specialists adapted to cold temperatures, high levels of solar radiation, and seasonally available water, together leading to oftentimes short growing seasons (Körner, 2003; Winkler *et al.* 2019a). As a result, rare and endemic species usually account for a substantial portion of high-elevation plant community richness (Casazza *et al.*, 2005, 2008). These species may be more susceptible to climate change as the main drivers of establishment and growth shift, negatively impacting existing populations and potentially preventing regeneration (Klanderud and Totland, 2005; Dickinson *et al.*, 2007; Schwartz *et al.*, 2006).

Upward shifts in high-elevation species ranges have already occurred and oftentimes coincide with a decrease in overall species richness (Pauli *et al.*, 2007; Chen *et al.*, 2011; but see Cannone and Pignatti, 2014; Rixen *et al.*, 2014). This is largely the result of evaporation-driven declines in available soil water as temperatures increase without a concurrent increase in precipitation (Elmendorf *et al.*, 2012; Wahren *et al.*, 2013). Increased temperature is predicted to enhance terrestrial productivity (Nemani *et al.*, 2003) but experimental evidence varies (Scheffers *et al.*, 2016), with some research suggesting that water-limited systems may not be able to respond positively unless warming-induced moisture stress is alleviated (Winkler *et al.*, 2016a). Some high-elevation species may be able to acclimate to changing conditions by shifting resource allocations to withstand stress (Theodose *et al.*, 1996; Gratani, 2014; Winkler *et al.*, 2016b) but the extent to which this is possible for alpine endemics or rare species remains largely unexplored (but see Ashton *et al.*, 2010; Graae *et al.*, 2011). Furthermore, it is unknown how population regeneration will be impacted by changing conditions experienced by long-lived perennial species (Williams *et al.*, 2015) and whether early life history strategies will determine specific responses to climate change (Pearson *et al.*, 2014; Salguero-Gómez *et al.*, 2016; Harrison and LaForgia, 2019). Determining the separate and combined effects of climate change on individual plant performance and survival can help infer how populations persist may be structured under future scenarios (Le Roux *et al.*, 2005; Thuiller *et al.*, 2008). Plant species that exhibit plastic morphologies and a suitable degree of physiological compensation may be better able to buffer populations from climate change, while those with relatively fixed life history strategies may experience population declines with little opportunity for regeneration (Franks *et al.*, 2014; Valladares *et al.*, 2014; Peterson *et al.*, 2018).

Making predictions and evaluating the level of susceptibility of endemic species to global change is further complicated when little is known about their basic biology and by the limited amount of research published on them (Casazza *et al.*, 2014; Gómez *et al.*, 2015). The high-altitude rock outcroppings that sit atop Sierra de San Pedro Mártir (SSPM) National Park in Baja California, México exist in a botanically rich area at the southern boundary of the California Floristic Province where relatively little ecological research has been carried out beyond natural history observations and research on the fire ecology of the park’s conifer species (Minnich *et al.*, 2000; Stephens *et al.*, 2003; Riemann and Ezcurra, 2005; Holmgren *et al.*, 2011; Rivera-Huerta *et al.*, 2016; Rebman *et al.*, 2018). Many of the species in SSPM have not been observed *in situ*, collected or deposited into herbaria in 30+ years (Riemann and Ezcurra, 2005), leaving little known about the current status of these species. Baja California contains approximately 3100 plant species of which 23.7% are endemic to the peninsula (Rebman *et al.*, 2016), with at least 66 species endemic to SSPM (Riemann and Ezcurra, 2005, 2007; Vanderplank *et al.*, 2018). Furthermore, SSPM possesses high levels of genetic isolation and environmental heterogeneity as a result of its abrupt topography relative to the surrounding Mediterranean and Sonoran desert systems often characteristic of the Baja California peninsula (Riemann and Ezcurra, 2007).

SSPM sits at the southern boundary of the California Floristic Province and is also located near the southern boundary of the subtropical jet stream, suggesting species in the park may be at increased risk of being negatively impacted by climate change as the jet stream and westerlies are expected to shift northward in response to warmer temperatures (Shindell *et al.*, 1999; Lu *et al.*, 2008). Historic climate reconstructions from tree-rings suggest that SSPM has experienced long, extreme drought events in the 1950s and has more recently experienced the single most intense drought year, 2007, since the tree-ring record began in 1658 (Meko *et al.*, 2013). Tree species in SSPM may be well equipped to tolerate climate period punctuated by extreme drought but the extent to which this translates to the smaller statured plant species is unknown. Additionally, temperatures are expected to increase 2–4 °C and precipitation is expected to decrease 30–50% in parts of Baja California by 2100 (Chen *et al.*, 2003; Cavazos and Arriaga-Ramírez, 2012; ). Furthermore, upward shifts in elevation distributions are predicted for species respond to increasing temperature (Lenoir *et al.*, 2008). This includes forecasts of large increases in plant diversity in and around SSPM (Loarie *et al.*, 2008). Together, these changes will surely have an impact on species already confined to the sky islands of SSPM, especially those restricted to the limited patches of alpine rock outcroppings there.

The cliff-dwelling cushion plant *H. brandegeei* (B.L. Rob. & Greenm.) Semple is a long-lived, perennial endemic to SSPM and only found on uncommon alpine outcroppings on the peaks of SSPM, making it a chasmophytic specialist (i.e. a plant growing in the crevices of rocks) with a narrow distribution and highly restricted range (Moran, 1969). This alone suggests this is a species worthy of concern and would likely be categorized at a high level of extinction risk (Thomas *et al.*, 2004; Dirnböck *et al.*, 2011). Little is known about *H. brandegeei* aside from locality information and descriptions of the species’ morphology based on type specimens (Robinson and Freeman, 1896; Moran, 1969). *H. brandegeei* may provide valuable insights into the potential responses of rare, high altitude species experiencing climate change, their ability to utilize micro-refugia, and how ranges may shift when upward migration is not an option. Furthermore, existing levels of phenotypic variation in the species may also buffer it from increasing stressors (Jump and Peñuelas, 2005; Aitken *et al.*, 2008) but this may only be temporary if climate change pushes populations past a tipping point beyond which compensation is improbable (Doak and Morris, 2010; Botero *et al.*, 2015). Thus, understanding early life history strategies and mortality rates during establishment may be suitable for identifying species’ regeneration niche while also predicting future population dynamics (Shimono and Kudo, 2003; Cochrane *et al.*, 2015).

In this study, we used environmentally controlled growth chambers to document the early life history responses of *H. brandegeei* to simulated climate warming and drought during its first growing season. We hypothesized that this high-elevation, long-lived, slow-growing, perennial cushion would decrease productivity in response to warming and show signs of drought stress in a warmer, drier climate. We characterized seed germination and survival of *H. brandegeei* during its first year of growth. We hypothesized *H. brandegeei* would undergo various morphological and physiological changes when exposed to stressors, including utilizing a higher WUE strategy and reducing aboveground growth. We quantified phenotypic trait variation and investigated responses in allocation to above (agb)- and belowground (bgb) structures to test morphological responses. We also measured responses in specific leaf area, WUE and carbon content of leaves to test leaf-level physiological responses. Our study also expands the known range distribution of *H. brandegeei* and provides data on the species in its native habitat.

## Materials and methods

### Study site and species

**Figure 1:**
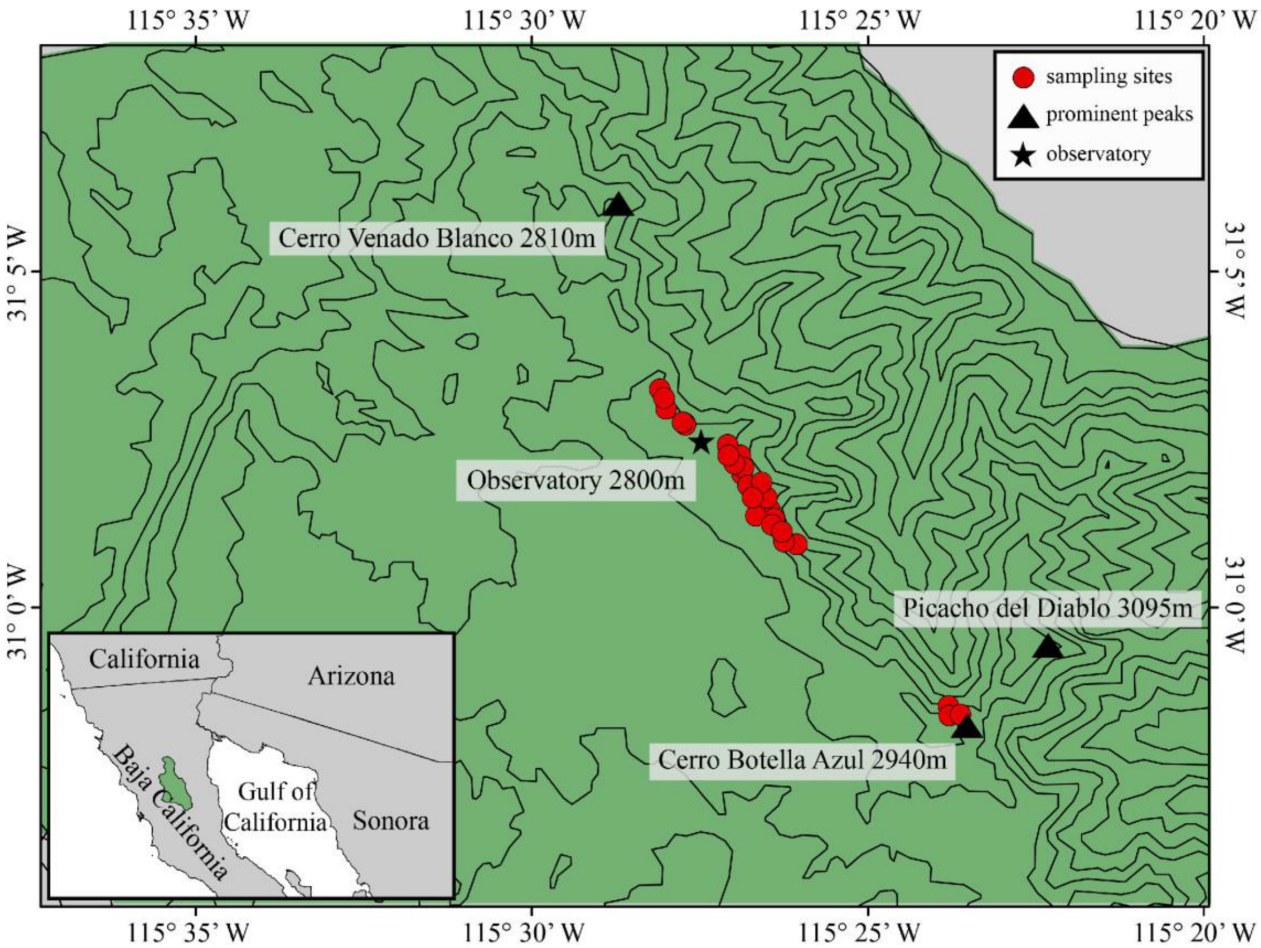
Contour map showing sampling locations (circles) of *Heterotheca brandegeei* in relation to prominent peaks (triangles) and México’s National Observatory (star) in SSPM National Park in Baja California, México (green polygon in inset).

SSPM National Park was founded in 1947 and is the fourth largest National Park in México (63 000 ha; Fig. 1). It is also the southern terminus of the Peninsular mountain ranges and, as a result, serves as the lower latitudinal boundary for many montane species (Minnich *et al.*, 1997; Riemann and Ezcurra, 2007; Burge *et al.*, 2016). SSPM contains the highest point of elevation in Baja California (Picacho del Diablo, 3096 m), intercepting northwesterly winds to create the Mediterranean climate on its western slopes and a rainshadow that gives way to Sonoran desert on its eastern slopes (Vanderplank *et al.*, 2018). SSPM also receives the highest amount of annual precipitation on the peninsula (approximately 600–700 mm; Hastings and Turner, 1965; Minnich *et al.*, 1997). The majority of substrate in the park is granitic, with soils currently un-described. The park’s granitic substrate also creates sparse alpine-like outcroppings where the sub-alpine conifer forest cannot persist and, instead, alpine chasmophytic specialists occur. A well-studied feature of SSPM is its un-managed fire regime history that make it one of the most pristine wildlands with intact mixed-conifer forests (Minnich *et al.*, 2000; Bojórquez-Tapia *et al.*, 2004; Skinner *et al.*, 2008). Few studies exists on the shorter-statured plants of SSPM beyond species descriptions, phylogenies and reporting of locality data (Moran *et al.*, 1969; Semple *et al.*, 1988; Thorne *et al.*, 2010; Simpson and Rebman, 2013; Rebman *et al.*, 2018).

**Figure 2:**
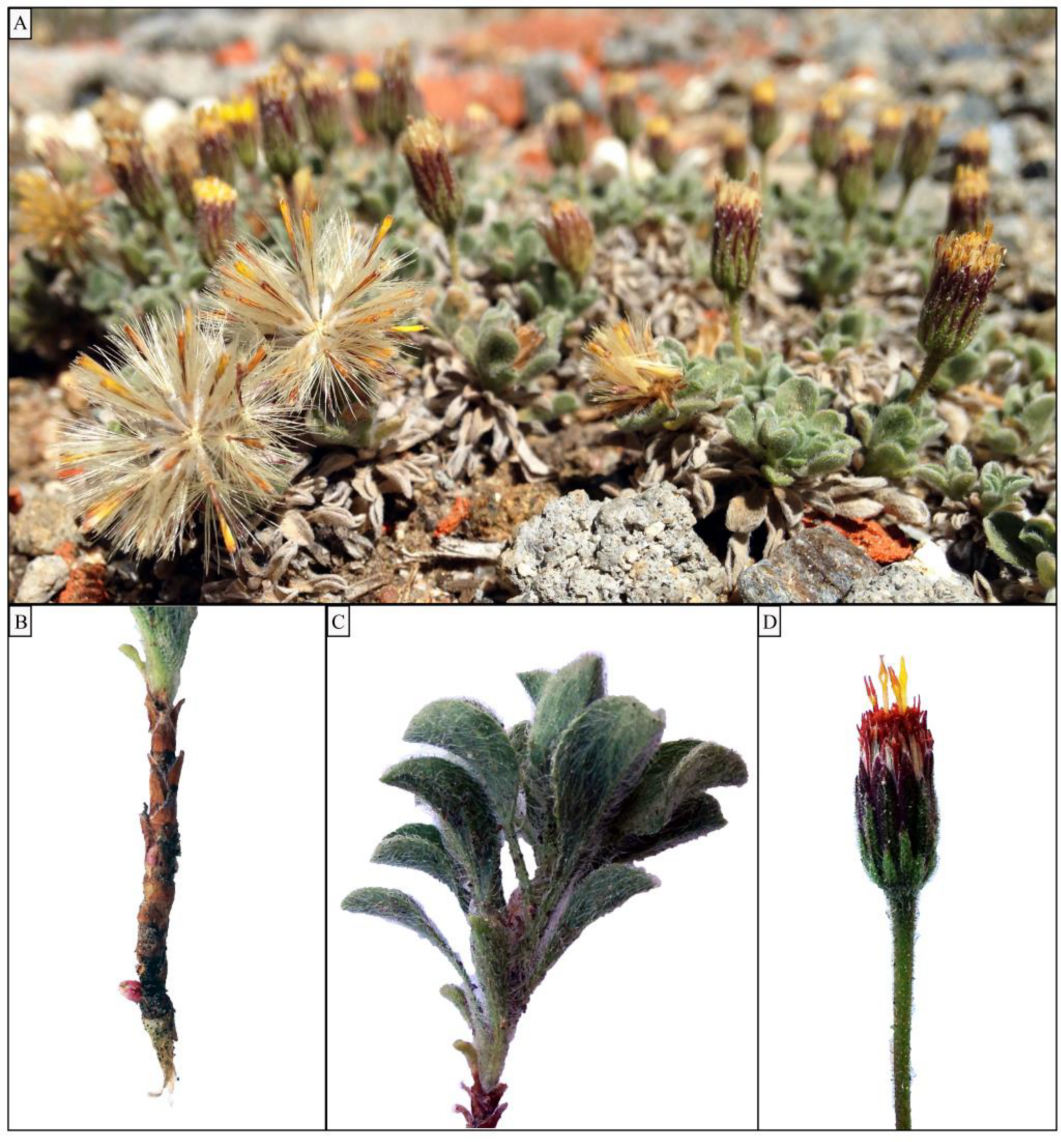
Morphological overview of *Heterotheca brandegeei* (**A**) in its habitat on a rock outcropping in SSPM National Park, (**B**) bgb structures including a prominent rhizome bud, (**C**) hirsute leaves as part of an agb rosette and (**D**) discoid flower head on a slender peduncle with few anthers and styles protruding.

*H. brandegeei* is a mat-forming, rhizomatous perennial first described in 1896 (Robinson and Freeman, 1896 as *Chrysopsis brandegeei* then as *H. martirensis* in Moran, 1969 and later reclassified in Semple *et al.*, 1988). The species was described as flowering May to September, with thick, hirsute, spatulate leaves growing in clusters close to the ground (Fig. 2). This trait combination is somewhat unique to the genus but most similar morphologically to the similarly rare, endemic *H. jonesii* in Utah (Welsh *et al.*, 1975). Semple *et al.* (1988) note that *H. brandegeei* is most similar to *H. viscida*, which occurs on cliff crevices in the Sky Islands of Southern Arizona through to West Texas. Solitary flowers sit on relatively slender, glandular peduncles up to 6 cm tall and produce disc achenes that are similar to related wind-dispersed asters (Fig. 2; Robinson and Freeman, 1896; Moran, 1969). The species was said to be common in crevices on flat granitic surfaces above 2800 m when it was described in 1969. *H. brandegeei* is common on rocks in full sun or partial shade from 2050 to 2800 m asl and, before this study, was known from only six sites (Moran, 1969; but see Rebman *et al.*, 2018). These rock outcroppings are often dominated by other endemics including *Stephanomeria monocephala*, *Sphaeromeria martirensis*, *Stenotus pulvinatus*, and non-endemics *Selaginella asprella*, *Potentilla wheeleri*, *Sedum niveum* and *Myriopteris wootonii* among others (Moran, 1969; Delgadillo, 2004; Thorne *et al.*, 2010). Communities in which *H. brandegeei* is found are more similar to damp meadows than to snowbed communities (Peinado *et al.*, 2005).

### Field sampling

We collected *H. brandegeei* seeds from 63 individual maternal plants from ten locations along a approximately 20 km transect in May 2014 (the beginning of the flowering period described in Moran 1969). We systematically surveyed rock outcroppings along the crest of SSPM as well as forested areas in between, revisiting historic localities while documenting new sites with GPS (Fig. 1). Seeds from individual plants were placed in separate coin envelopes and stored in a dry, low humidity environment (ca. 10% RH) until experimentation began the following year.

We also measured site-level characteristics for a subset of locations where *H. brandegeei* were observed, regardless of seed availability, in an effort to expand knowledge of this species in its natural habitat in SSPM. For 12 individual plants, we measured the maximum width of the plant (cm), azimuth (°) and slope (°) of the rock outcropping where each individual occurred. Since it was often difficult to delineate individual plants, we measured the entire length of individuals that formed a continuous mat in a given fissure and only collected seeds from within this measured area. We recorded phenological observations across these sites and noted whether individuals within a site had flower buds present, were flowering, had seeds present or had already set seed. We also noted species in proximity of the focal individual.

### Laboratory methods

We first soaked *H. brandegeei* seeds in a 1% bleach solution for 3 min prior to sowing to decrease the occurrence of mould. We initially ran germination trials in growth chambers to infer optimal requirements for germination by replicating typical conditions the seeds would experience in SSPM if they germinated early in the spring after snowmelt (8.1 °C during the day and 1.0 °C at night on a 12:12 day:night cycle), during the early summer (13.2 °C during the day and 6.0 °C at night on a 14:10 day:night cycle), or during late summer when monsoonal precipitation reaches its peak (17.6 °C during the day and 11.9 °C at night with a 13.5:10.5 day:night cycle; Douglas *et al.*, 1993; UNAM, 2017). After 2 weeks, no germination had occurred in either the early spring or early summer chambers but seedlings had emerged in late summer conditions, which matched conditions in the UC Irvine greenhouse in May–June when this experiment began. Thus, to maximize space, 1200 *H. brandegeei* seeds were sown in the UC Irvine greenhouse into individual 5 × 5 × 8-cm containers filled with a custom made 3:3:2:3 soil mix of redwood chips:dried moss:sand:perlite. Seeds were buried in the top 2 cm of soil and were watered regularly to keep containers moist until germination occurred and seedlings established up to 1 month. The ten sampling sites and maternal lineages were randomly placed on greenhouse benches, and containers were randomly rotated weekly to control for potential spatial heterogeneity in conditions in the greenhouse. Greenhouse temperatures during germination and the first month of growth were similar to August temperatures in SSPM (averaging 18 °C daily and 12 °C nightly in the greenhouse; UNAM, 2017). We chose a greenhouse in part not only due to constraints on conducting such an experiment in the field but also in an effort to more rapidly and accurately simulate climate change conditions and reduce variability in biotic and abiotic factors that may have reduced treatment effects (Gibson *et al.*, 1999).

Seedlings were transplanted to larger containers (12 × 13 × 12 cm) after 1 month in the greenhouse and assigned to treatment conditions in growth chambers. We randomly assigned 30 individuals to each of four treatments (*n* = 120): ambient control, warming, drought and warming + drought (W + D). Treatments were carried out in experimental growth chambers, and plants were rotated weekly to account for potential small-scale variation in chambers. Ambient temperature and precipitation levels were determined using historical climate data for August averaged over 7 years (2007–2014; UNAM, 2017). All chambers were set to a 13-h light and 11-h dark cycle that also matched August cycles in SSPM (UNAM, 2017). The control chamber was set to simulate late summer growing season conditions in SSPM and was set to a daytime temperature of 17.6 °C and a night-time temperature of 11.9 °C. We simulated ambient precipitation by watering plants with 20 mm of water weekly (ambient and warming treatments). Heating treatments simulated a predicted +4 °C temperature increase for the region of Baja California that includes SSPM (Cavazos and Arriaga-Ramírez, 2012), and chambers were set to 21.6/15.9 °C (day/night; warming and W + D treatments). Drought treatments followed the highest predictions of a 50% decrease in mean annual precipitation, and plants received 20 mm of water every other week (drought and W + D treatments; Cavazos and Arriaga-Ramírez, 2012).

### Measurements

We measured phenological, morphological and physiological traits to capture treatment responses of individuals and summarized the natural history of this unstudied species. We chose traits that have previously been shown to be sensitive to environmental changes experienced by other alpine cushion species (Yang *et al.*, 2011; Soudzilovskaia *et al.*, 2013; Spasojevic *et al.*, 2013; Winkler *et al.*, 2016a). Phenological traits included time to germination and leaf expansion of up to the first five true leaves during the establishment phase in the greenhouse. We surveyed daily for germination once seeds were sown and tracked individual plants until mortality occurred or plants were harvested for measurements.

We measured morphological traits throughout the experiment to test individual stress responses in each treatment and quantify phenotypic trait variation across maternal lineages and sites. We harvested two individuals from each treatment every other week for 120 days or until no plants remained in growth chambers. We cut stems at the soil surface and sorted plant parts into leaves and stems. All leaves were counted, weighed and digitally scanned with a Canon MF8200C printer (Canon, Tokyo, Japan). We calculated leaf area for all leaves (maximum number measured was 74 leaves) using ImageJ v. 1.8.0_112 (Schneider *et al.*, 2012). We weighed the five largest fresh leaves for specific leaf area (SLA) measurements. Belowground root biomass was excavated and sieved to remove soil using a no. 30-mesh sieve pan. Roots were then washed in water baths, sieved again and rewashed. All biomass was dried for 48 h at 60 °C to obtain dry mass of agb, bgb and total biomass. We calculated individual and average SLA using fresh and dry weight values obtained for the five measured leaves. Additional metrics calculated using the above-mentioned data are reported in Supplemental Table S2. These include: total, average, minimum and maximum leaf area, stem dry weight, total and average leaf dry weight, total root length, average specific leaf area for each of the five largest leaves, and maximum and minimum specific leaf area.

Lastly, we measured leaf chemistry including ^13^C and leaf carbon (*C*_mass_). Leaf ^13^C and *C*_mass_ were analyzed at the University of California, Davis Stable Isotope Facility via an elemental analyser interfaced to a mass spectrometer (PDZ Europa, ANCA-GSL and PDZ Europa 20–20, Secron Ltd, UK). We converted carbon isotope ratios to discrimination values (*Δ*, per mil—a time-integrated measure of WUE (Farquhar *et al.*, 1989; Dawson *et al.*, 2002). Lower values of *Δ* indicate higher intrinsic WUE values (Dawson *et al.*, 2002). We also converted *C*_mass_ values to reflect the % carbon content of leaves using the dry weight of leaf tissues sampled and measured *C*_mass_.

### Statistical methods

We visualized germination patterns using dose–response curve fitting to model the proportion of seeds that germinated through time. Data were fitted to a three-parameter log-logistic regression model using the drc package in R (Ritz and Streibig, 2005). We explored the relative contributions of sampling locations, maternal lines and individual seeds in explaining variances in the number of days until germination occurred with a linear mixed effects model. Location, maternal line and individual plant ID were included as nested random effects in an intercept-only model. We then extracted the variance components from the model using the VarCorr function in the lme4 package (Bates *et al.*, 2011). Variance components were then calculated following Crawley (2012) where each variance component (*σ*^2^) is a proportion of the sum of the standard deviations. We followed this same procedure to explore variation in the phenological timing of leaf emergence for the first true leaf. We calculated these values as the number of days since a given individual had germinated. Metrics for the four additional leaves measured for each plant are reported in Supplemental Table S2.

Next, we compared linear mixed effects models to determine the most appropriate combination of factors that predict treatment responses in our experiment (Aho *et al.*, 2014). Models included warming, drought and their interaction as fixed effects, and plant age as a covariate to control for potential effects of age on the response variables. We included the site, where seeds were collected, maternal line and individual plant ID as random effects to account for pseudo-replication across sites and maternal lineages. We also included plant ID as a random effect nested within maternal lineages (Harrison *et al.*, 2018). We tested the predictive ability of each of the main effects by comparing the Akaike Information Criterion scores corrected for small sample sizes (AIC_c_) of the full model to simpler versions (Johnson and Omland, 2004; Aho *et al.*, 2014). We used *Δ*AIC_c_ to calculate Akaike weights (*w_i_*) as the relative likelihood that a given model was the best. For the best model, we calculated marginal *r*^2^ to estimate the predictive power of explanatory variables and conditional *r^2^* to estimate the total predictive power of fixed and random effects together (Nakagawa and Schielzeth, 2013). We tested all variables for pairwise correlation across the study and retained variables that had correlation coefficients under |0.75| (Supplemental Table S1). When variables were highly correlated, we retained those variables related most directly to our hypotheses. Thus, we did not test abg or b biomass as these were strongly correlated with total biomass and this was suitable for testing our primary hypothesis. We built separate models to test treatment responses in total biomass, number of leaves, leaf carbon content, average specific leaf area of the five largest leaves and leaf-level WUE (*Δ*). We also tested treatment effects on plant mortality using a logistic mixed effects model with mortality, warming, drought and their interaction as binary fixed effects, and location, maternal line and individual plant ID as random effects.

The best model for each response variable was subsequently used to test treatment effects. We did this by employing the ANOVA function in the lmerTest package (Kuznetsova *et al.*, 2017) with Satterthwaite approximations for degrees of freedom. We again extracted the variance components from each model to determine how much of the observed variation in each response variable could be attributed to maternal effects (environmental, genetic or error) from locations and maternal lines that seeds were collected from. To test fixed effects for our mortality model, we used a type-III ANOVA using Wald χ^2^ tests in the car package (Fox and Weisberg, 2011). Finally, we used *post hoc* pairwise comparison tests and accounted for multiple comparisons by adjusting *P* values with the Holm method to determine significance among treatment groups using the lsmeans package in R (Lenth, 2017). We did this for all variables used in mixed models as well as agb and bgb biomass in order to explore potential variable responses to treatments. All mixed models were built using the nlme package in R 3.3.2 (Pinheiro *et al.*, 2018; R Core Team, 2014), except the mortality model that was built using the lme4 package (Bates *et al.*, 2011).

## Results

### Habitat characteristics and field observations

Plants were typically found on the northeasterly exposures with an average azimuth of 91.64 ± 11.18° and on granite outcroppings with 55.77 ± 7.92° slopes. Most *H. brandegeei* plants were flowering, while fewer had not produced flower buds yet or had already set seed. Individual plants produced anywhere from 1 to 142 flowers (median = 22, mean = 24.5 ± 1.92 SEM). *H. brandegeei* cushions had a maximum width of 20 ± 2.87 cm (mean ± SEM). This included cushions that spanned large portions of both shaded and exposed crevices and fissures (Supplemental Figure S1). Other species that were frequently observed in associated with *H. brandegeei* included *S. monocephala*, *P. wheeleri, S. asprella, S. niveum, S. martirensis, M. wootonii* and *S. pulvinatus*.

### Greenhouse germination and leaf phenology

**Figure 3:**
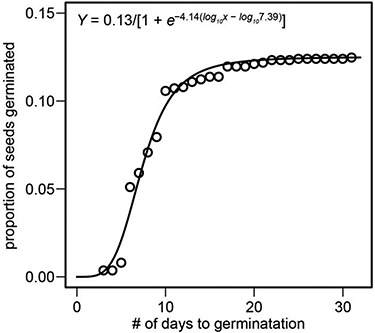
Cumulative proportion of *Heterotheca brandegeei* seeds germinated through time in greenhouse conditions. Data were fitted to a three-parameter log-logistic regression model. Points represent the cumulative proportion of germinated seeds at each time step (days) since seeds were sown at day 0.

In total, 171 *H. brandegeei* seeds germinated from 16 maternal lines and six locations. Time-to-germination was 8.85 days ±0.33 (mean ± SEM; Fig. 3; Table S3). Germination rates began to decline approximately 10 days after seed sowing (Fig. 3). Most of the variation in time-to-germination was explained by residual error (*σ*^2^ = 0.71; Table S4), though differences between individuals within maternal lines also explained a relatively high amount of variation (*σ*^2^ = 0.19). This suggested maternal lines contributed to the variation observed in germination timing across the seeds a given line produced in SSPM. Differences between maternal lines or locations explained smaller amounts of variation (between maternal lines: *σ*^2^ = 0.04; between locations: *σ*^2^ = 0.05; Table S4). The emergence of the first true leaf occurred 8.07 days ±0.23 after seeds had germinated (Table S3). Variation in leaf emergence was explained by differences between individuals within maternal lines (*σ*^2^ = 0.15; Table S4) and between maternal lines (*σ*^2^ = 0.05), though most of the variation was residual error (*σ*^2^ = 0.81). None of the observed variation was explained by sampling location (*σ*^2^ = 0.00; Table S4).

### Trait responses to warming and drought

Total biomass and leaf traits (# of leaves, SLA, % C in leaves, WUE) were best predicted by the interaction of warming and drought treatments with plant age as a covariate (marginal *r^2^* = 0.33–0.57; Tables S5–S9). Models predicting number of leaves without the interaction or plant age scored nearly as well (Table S6), but across all response variables, models without treatment were always worse (*Δ*AIC_c_ = 7.30–69.64).

**Figure 4:**
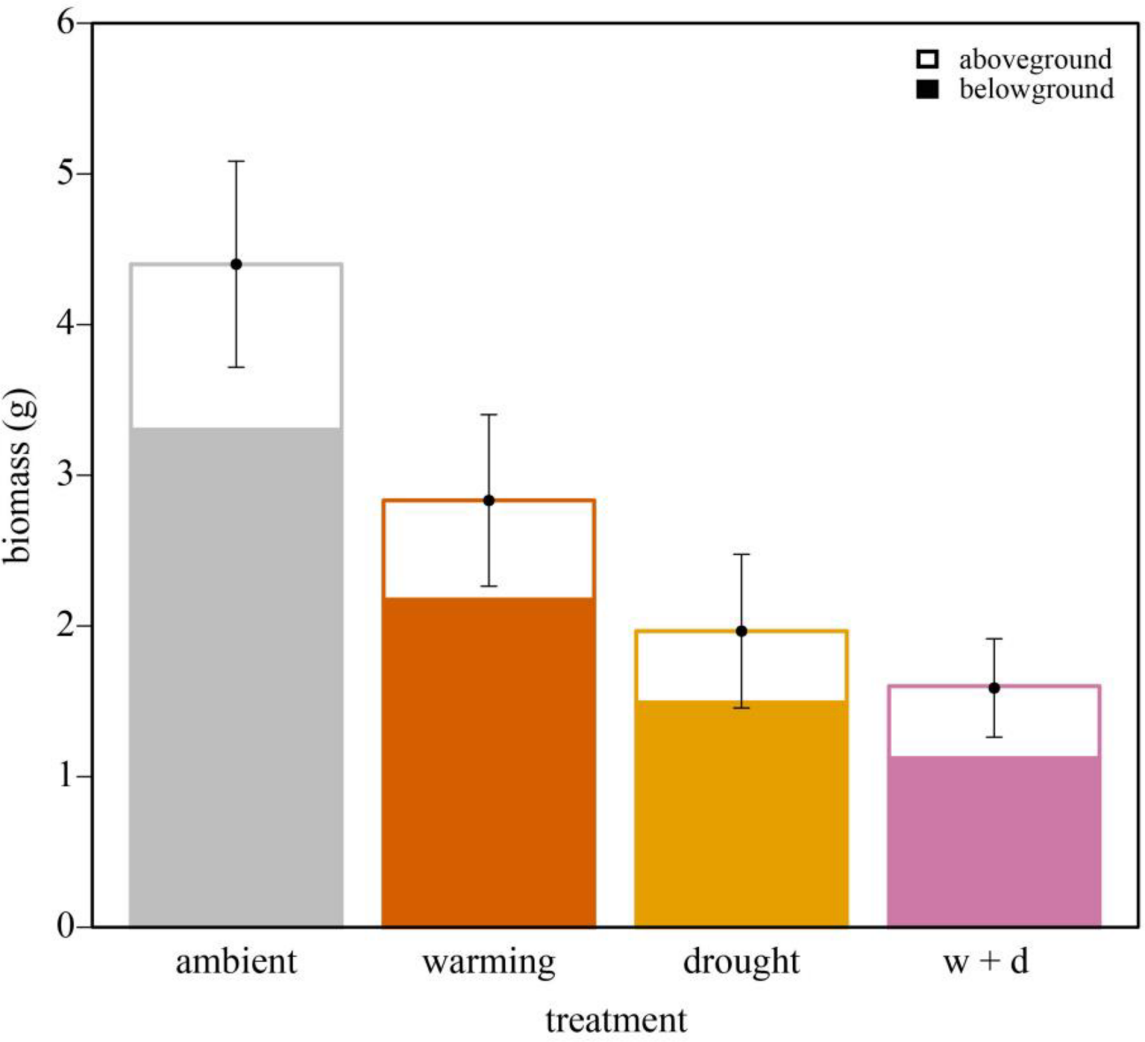
Total biomass responses of *Heterotheca brandegeei* to warming and drought treatments (grey = ambient, orange = warming, yellow = drought, pink = warming + drought). Upper hollow portion of each stacked bar represents agb biomass (g) and lower filled portion represents bgb biomass (g). Mean total biomass and SEM bars are denoted for each treatment.

Aboveground biomass declined 60% in response to drought treatments, while warming (W) and W + D only had a minor effect on agb biomass (Fig. 4, Table 2). Similarly, bgb biomass only responded to drought (Fig. 4; Table 2), with a 55–66% decline in root biomass in drought and W + D treatments relative to ambient conditions. Total biomass responded to warming (*F*_1,7_ = 5.87, *P* = 0.046) and drought (*F*_1,7_ = 20.57, *P* = 0.003) but not to the interaction of the two (*F*_1,7_ = 2.34, *P* = 0.170; Table 1). Overall, total biomass declined by 55–75% in drought and W + D treatments relative to ambient conditions (Fig. 4; Table 2). Plant age helped to explain total biomass responses (Table 1). Marginal *r^2^* (variance explained by fixed effects) and conditional *r^2^* values (variance explained by fixed and random effects) were similar for the final total biomass model, suggesting treatments and plant age explained nearly all of the variation in biomass measurements. However, random effects did provide some additional explanatory power (2%; Table 1), with a majority of it being attributed to error (*σ*^2^ = 0.97; Table 3).

**Table 1:** Linear mixed effects model results for the best-supported models with fixed effects including warming, drought, the interaction of the two (w × d) and plant age. *F* statistics and degrees of freedom are reported with *P* values. Values showing significant differences at *α* = 0.05 are shown in bold font. Marginal and conditional *r^2^* are reported for each model

	Warming		Drought		w × d		Plant age				
	*F*	*P*	*F*	*P*	*F*	*P*	*F*	*P*	df	marg. *r^2^*	cond. *r^2^*
Total biomass (g)	5.87	**0.046**	20.57	**0.003**	2.34	0.170	29.54	**0.001**	1,7	0.57	0.59
# leaves	10.25	**0.015**	12.48	**0.010**	2.01	0.199	9.39	**0.018**	1,7	0.43	0.62
SLA (mm^2^ mg^−1^)	1.11	0.328	3.02	0.126	0.07	0.780	34.38	**<0.001**	1,7	0.45	0.45
% C in leaves	1.59	0.254	7.42	**0.034**	0.01	0.930	2.63	0.156	1,6	0.22	0.95
WUE (∆)	2.58	0.159	1.25	0.306	25.02	**0.002**	51.35	**<0.001**	1,6	0.33	0.98

**Table 2:** Biomass and leaf-level trait means (± SEM) at the end of the 120 days of experiment in ambient, warming, drought and W + D treatments. Sample sizes (*n*) are also provided. Subscript letters indicate differences based on pairwise comparisons at *α* = 0.05

	Ambient	*n*	Warming	*n*	Drought	*n*	W + D	*n*
agb (g)	1.10 ± 0.14_a_	12	0.66 ± 0.16_ab_	11	0.45 ± 0.08_b_	11	0.48 ± 0.11_ab_	9
bgb (g)	3.30 ± 0.59_a_	12	2.17 ± 0.45_ab_	11	1.49 ± 0.43_b_	11	1.12 ± 0.24_b_	10
Total biomass (g)	4.40 ± 0.68_a_	12	2.83 ± 0.57_ab_	11	1.97 ± 0.51_b_	11	1.70 ± 0.33_b_	9
# leaves	41.50 ± 5.81_a_	12	22.09 ± 5.45_ab_	11	19.18 ± 4.00_b_	11	11.70 ± 1.70_b_	10
SLA (mm^2^ mg^−1^)	17.37 ± 1.81_a_	12	18.56 ± 2.61_a_	11	20.11 ± 3.19_a_	11	23.60 ± 4.12_a_	8
% C in leaves	46.91 ± 2.48_a_	12	49.51 ± 3.41_a_	10	54.13 ± 4.40_a_	12	50.29 ± 5.61_a_	7
WUE (∆)	30.02 ± 0.77_a_	6	29.01 ± 0.87_a_	5	28.63 ± 0.83_a_	6	34.27 ± 2.78_b_	4

**Table 3:** Variance (*σ*^2^) explained by nested random effects from best-supported linear mixed effects models. Variance components include the amount of variation explained by differences between locations, between maternal lines within locations, between individuals within maternal lines, and by residual error

	Between locations	Between maternal lines within locations	Between individuals within maternal lines	Residual error
Total biomass (g)	0.03	0.00	0.00	0.97
# leaves	0.00	0.00	0.34	0.66
SLA (mm^2^ mg^−1^)	0.00	0.00	0.00	1.00
% C in leaves	0.00	0.10	0.84	0.06
WUE (∆)	0.00	0.95	0.00	0.05

**Figure 5:**
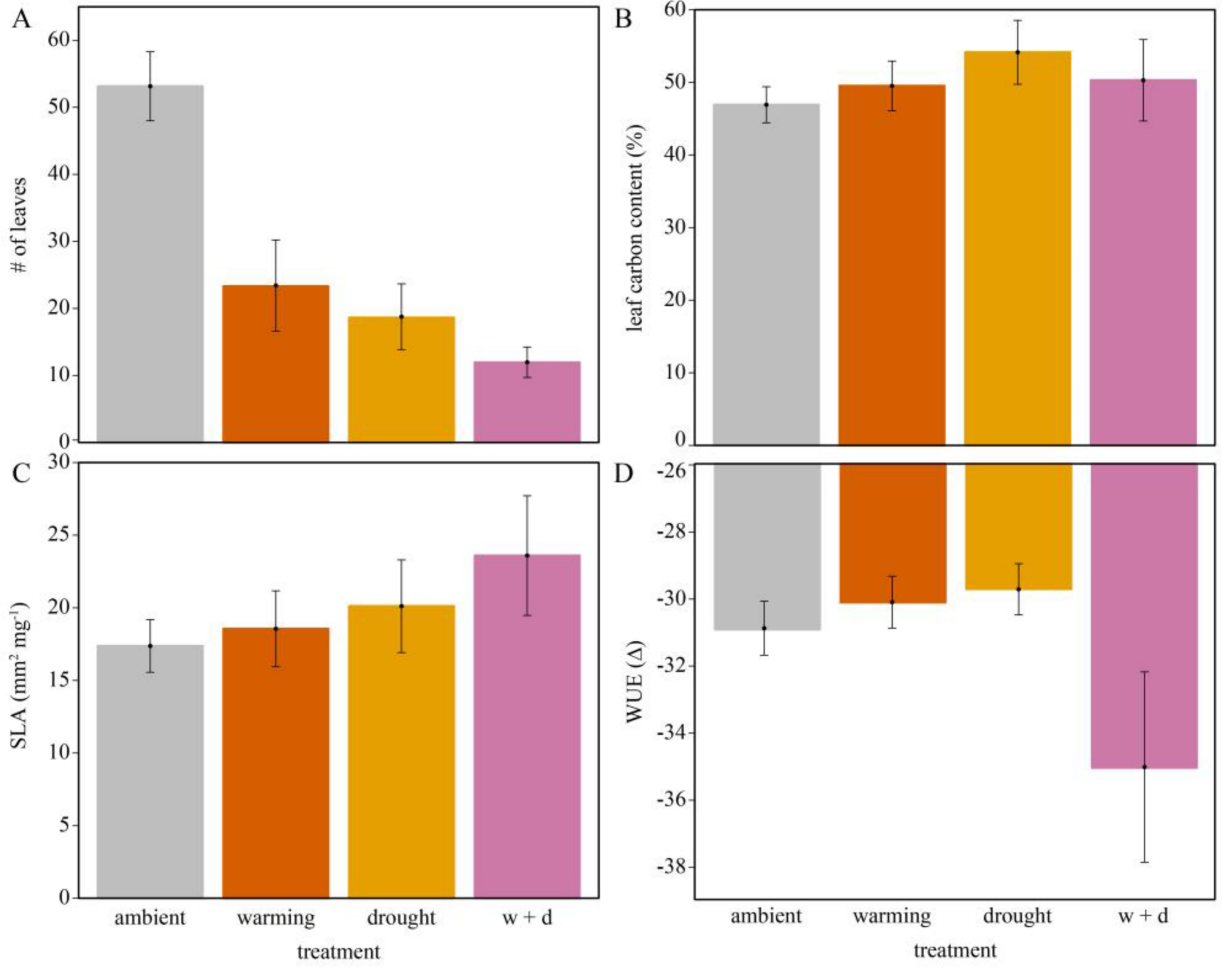
Leaf-level responses to warming and drought treatments including (**A**) the number of leaves produced, (**B**) leaf carbon content (%) in leaf tissue, (**C**) SLA (mm^2^ mg^−1^) and (**D**) intrinsic WUE (*Δ*). Mean values and SEM bars are denoted for each treatment (grey = ambient, orange = warming, yellow = drought, pink = warming + drought).

Corresponding to changes in biomass, plants produced 54–72% fewer leaves in drought and W + D treatments relative to ambient conditions (Fig. 5, Table 2). Both the individual effects of warming (*F*_1,7_ = 10.25, *P* = 0.015) and drought on the number of leaves were significant (*F*_1,7_ = 12.48, *P* = 0.010), but the interaction of the two treatments was not (*F*_1,7_ = 2.01, *P* = 0.199; Table 1). Random effects explained additional variance compared to only fixed effects (conditional *r^2^* = 0.62; marginal *r^2^* = 0.43; Table 1). A majority of the variation was explained by error (*σ*^2^ = 0.66) but variability between individuals within maternal lines also explained a portion of the observed variance (*σ*^2^ = 0.34; Table 3), suggesting that genetic variance or maternal effects partially explain treatment responses.

The average specific leaf area of the five largest leaves produced by each plant remained unchanged in warming (*F*_1,7_ = 1.11, *P* = 0.328), drought (*F*_1,7_ = 3.02, *P* = 0.126), and W + D treatments (*F*_1,7_ = 0.07, *P* = 0.780; Fig. 5, Tables 1–2). Plant age was the only predictor to explain specific leaf area across treatments (*F*_1,7_ = 34.38, *P* < 0.001; Table 1). This makes sense, considering that plant age is a typical driver of leaf size, and likely overshadowed treatment effects. Random effects did not improve the explanatory power of this model (both marginal and conditional *r^2^* = 0.45; Table 3).

The % carbon content of leaves increased in response to drought treatments (*F*_1,6_ = 7.42, *P* = 0.034; Fig. 5), but effect size was relatively small, such that pairwise *t*-tests across treatments were not significant (Table 2). Percent carbon content of leaves was the only trait that plant age did not help predict (Table 1), suggesting the warming and drought treatments accounted for most of the variance explained by the fixed effects (marginal *r^2^* = 0.22). However, the amount of variance explained was more than twice as much when both fixed and random effects were considered (conditional *r^2^* = 0.95). Most of the variance in % carbon content of leaves was explained by variation among individuals within maternal lines (*σ*^2^ = 0.84; Table 3). Additional variability was explained by differences between maternal lines within locations (*σ*^2^ = 0.10; Table 3), suggesting microhabitat differences experienced by maternal lines may have influenced how individuals responded to treatments.

Leaf-level WUE did not respond to warming (*F*_1,6_ = 2.58, *P* = 0.159) or drought (*F*_1,6_ = 1.25, *P* = 0.306) but did respond to their interaction (*F*_1,6_ = 25.02, *P* = 0.002; Table 1). WUE decreased in the W + D treatment by 12% relative to ambient (Fig. 5, Table 2) but was largely explained by random effects (conditional *r^2^* = 0.98) especially maternal line (*σ*^2^ = 0.95; Table 3). This further suggests that maternal influence, in part, predicted the ability of plants to adjust WUE in response to warming and drought.

### Mortality responses to warming and drought

**Figure 6:**
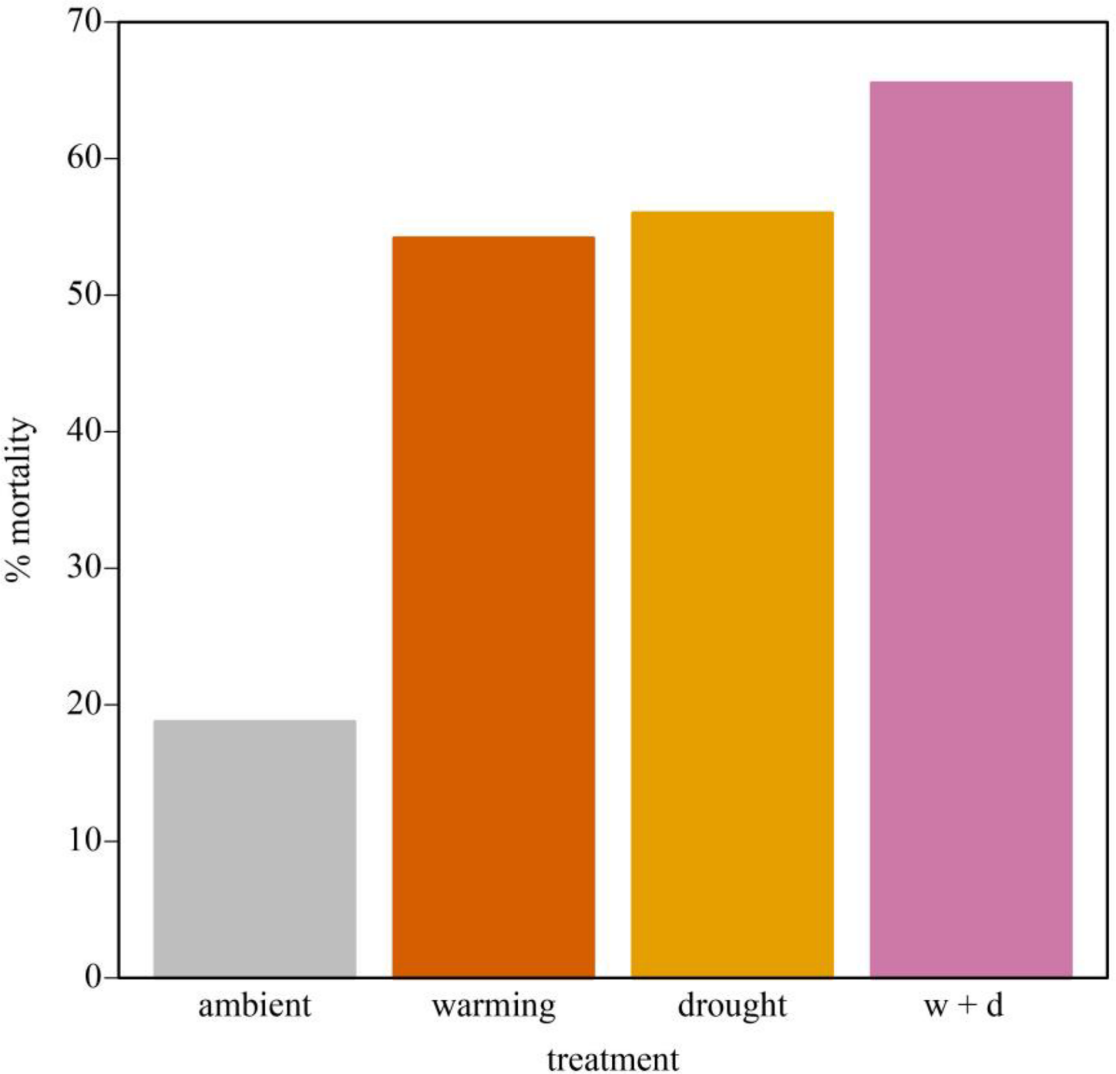
Total % mortality of *Heterotheca brandegeei* individuals in each treatment (grey = ambient, orange = warming, yellow = drought, pink = warming + drought). Final mortality was calculated after 120 days of treatment or until no plants remained in growth chambers.

Seedling mortality was prevalent across all treatments (Fig. 6), with approximately 30% of seedlings dying during the experiment. Seedling mortality was best predicted by a model without an interaction of fixed effects (*w_i_* = 0.53), but the full model had nearly the same AICc value (*Δ*AIC_c_ = 1.23; Table S10). The effects of drought (χ^2^ = 5.97; *P* = 0.02) and warming treatments (χ^2^ = 4.83; *P* = 0.03) lead to higher mortality rates relative to ambient conditions (Fig. 6). Approximately, 19% of individual plants died under ambient conditions whereas 54–56% of plants died under the individual warming and drought treatments (Fig. 6). W + D saw the highest mortality with 65% of individuals dying before the experiment ended.

## Discussion

The ability of rare, endemic populations to regenerate in the face of climate change is poorly understood both from the perspective of biological constraints on performance and from the geometry of the environmental template. This is particularly true in high-altitude biodiversity hotspots like SSPM National Park in Baja California, México. There, fragmented alpine outcroppings dot mountain peaks and contain plant communities composed of chasmophytic specialists of which little is known beyond species descriptions and sparse locality data. We tested if phenotypic variation in early life history strategies may buffer populations of the cushion *H. brandegeei* by exposing seedlings to experimental warming and drought conditions. Overall reductions in biomass corresponded to reduced investment in photosynthetic surfaces, which likely served to reduce potential water stress, but constrained plant size. Individuals maintained high specific leaf area and similar carbon concentrations in leaves across treatments, further suggesting that plants were able to at least temporarily respond to the negative effects of treatments by altering morphologies. Individual plants exhibited decreased leaf-level WUE in response to combined warming and drought treatments but maintained relatively high WUE in response to warming and drought separately, suggesting that the species may be able to tolerate warming or drought but not both. Seedling mortality was three times higher in warming and drought treatments, further suggesting that *H. brandegeei* will likely experience population declines as new seedlings fail to persist in a warmer, drier climate. Overall, these phenotypic adjustments may be enough to enable the species to response to climate change in the short term, but the overall impacts of climate change at the population-level may not be enough to persist across longer timescales.

Shifting how biomass is allocated during the seedling establishment phase can be crucial for survivorship (Lloret *et al.*, 1999; Harrison and LaForgia, 2019). Seedling plasticity, however, can be limited by maternal investment, epigenetic effects and environmental stress imposed by future climate conditions (e.g. Lazarus *et al.*, 2018). Although *H. brandegeei* produced fewer leaves in individual and combined warming and drought treatments, the leaves they did produce were similar in specific leaf area and % of carbon compared to ambient treatment. We also found that *H. brandegeei* seedlings were able to maintain high WUE in response to the individual warming and drought treatments, which matched the compensating response in specific leaf area and carbon content. However, this relationship broke down in the combined warming and drought treatments, where *H. brandegeei* WUE was lower than ambient and warming and drought treatments. This matches expectations that intrinsic WUE decreases with increasing evaporative demand (Körner *et al.*, 1991; Lloyd and Farquhar, 1994). The effect was likely caused by reduced control of stomata at this level of stress. Furthermore, WUE in warming and drought treatments was likely compensated by structural changes (i.e. fewer leaves). This further reflects how phenotypic variability captured within (*σ*^2^ = 0.34 for # of leaves; *σ*^2^ = 0.84 for % C in leaf tissue) and between (*σ*^2^ = 0.95 for WUE) maternal lines shapes trait responses.

*H. brandegeei* reduced investment in the quantity of leaves but maintained the quality. Producing fewer leaves, with on average slightly elevated percentages of carbon, may be an attempt to increase the lifespan of a leaf as it responds to treatment stressors (Casper *et al.*, 2001). Indeed, for at least some species, maintaining fewer, stable leaves can be a successful drought-avoidance strategy (Escudero *et al.*, 2008). This trait variability is the most likely mechanism by which plants survived warming and drought treatments. These results could be used to infer demographic processes that may allow *H. brandegeei* to maintain populations in an already highly variable environment (Morris and Doak, 2004).

Mortality was high in all warming and drought treatments. Recent work suggested that increased root length was the only trait that predicted seedling survival in response to drought in an annual grassland (Harrison and LaForgia, 2019). We found no change in root length in response to drought treatments (Supplemental Table S2) and bgb allocation declined overall, suggesting *H. brandegeei* bgb strategies may be relatively fixed compared to leaf strategies. This could be attributed to the specialized root system of *H. brandegeei*, which is adapted to outcrop crevices and fissures (Houle and Phillips, 1989) and relatively variable agb conditions ranging from fully exposed surfaces to nooks shaded by rock ledges (Supplemental Figure S1).

Drought is already having marked impacts on plant populations across the southwestern USA and northeastern México (Miriti *et al.*, 2007; Bullock *et al.*, 2010; Winkler *et al.*, 2018, 2019b; but see Peters *et al.*, 2012). Individual plants may be able to tolerate short-term drought by adjusting strategies, as we demonstrated here, but these adjustments may not be enough to compensate for the negative population effects brought by increased plant mortality within a population (e.g. Ogle and Reynolds, 2004; Le Roux *et al.*, 2005). For example, alpine grass species in Switzerland were able to maintain agb production in response to experimental short-term warming when adequate soil moisture was maintained, but experienced biomass declines and mortality when short-term warming coincided with drought (De Boeck *et al.*, 2016). High levels of alpine plant mortality were also observed in response to natural drought in Australia, suggesting that the seasonal timing of drought events may be more important than their duration (Griffin and Hoffmann, 2012). Timing of seasonal precipitation plays a major role in Baja California systems (Douglas *et al.*, 1993) and has shaped the fire history of SSPM (Skinner *et al.*, 2008). We demonstrate that drought during the seedling establishment phase can reduce survival rates of *H. brandegeei.* Surely, the timing of drought in systems like the sky islands of SSPM will not only influence establishment, but also phenological traits and performance of already established individuals (Crimmins *et al.*, 2011; present study). These patterns share similarities with how the sub-Antarctic cushion *Azorella selago* responds to experimental drought (Le Roux *et al.*, 2005). Reduced rainfall treatments caused increased stem mortality and accelerated senescence. Our study also showed increased mortality, albeit on single-stemmed seedlings. The response of older, multi-stemmed *H. brandegeei* would likely have produced results similar to *A. selago*, whereby individual plants were unable to support biomass at current levels and, thus, lose individual stems to compensate for reduced water availability (Le Roux *et al.*, 2005; Barbeta *et al.*, 2013).

Additionally, increased temperature can compound the effects of water-deficit stress (i.e. drought; De Boeck *et al.*, 2016). High-altitude cushions like *H. brandegeei* can tolerate intense solar radiation and exposure to high temperatures (Cavieres *et al.*, 2006; Kleier and Rundel, 2009; Graham *et al.*, 2012), but only until heat-tolerance limits are met, beyond which cellular damage or mortality occurs (Neuner *et al.*, 2000). At the same time, *H. brandegeei* has characteristic features shared with alpine cushion species around the globe (Fischer and Kuhn, 1984; Cavieres *et al.*, 2006; Kleier and Rundel, 2009; Sklenář *et al.*, 2016). These include hirsute leaves and a prostrate growth that enables individuals to withstand intense daily temperatures that also likely capture heat to prevent damage when seasonal or night-time temperatures drop near freezing.

Cushion and mat-forming species such as *H. brandegeei* create microclimates that promote their own growth, and likely the growth of other plant species (Reid *et al.*, 2010; Kleier *et al.*, 2015). This is especially important in many alpine and rock outcrop communities where suitable micro-habitat created by cushion and mat-forming plants facilitate their own productivity while increasing community diversity (Reid *et al.*, 2010). This results in relatively low ratios of agb:bgb competition (Wiser *et al.*, 1996; Lavergne *et al.*, 2003) and also potentially increases the need for physiological integration, or sharing resources among stems within an individual (Roiloa *et al.*, 2014). We found that variation between maternal lineages or individuals within lineages helped explain responses to warming and drought (e.g. number of leaves produced, % C in leaves and WUE). This level of phenotypic variation created by maternal lineages may buffer *H. brandegeei* from the negative impacts of climate change, as has been shown to be the case in the alpine cushion *Silene acaulis* (Peterson *et al.*, 2018).

Some of the between-maternal-line differences could be attributed to micro-habitat differences that were not measured (Frei *et al.*, 2012). It is also possible that the same maternal lineage was unintentionally sampled multiple times within a site and presumed to be separate maternal lineages. For example, Liu *et al.* (2007) found the cliff-dwelling herb *Oxyria sinensis* has ramets that can occupy an area of 7–9 m and can be separated by distinct individuals that form their own patchwork of ramets, exploiting patchy resources within bedrock fissures (Liu *et al.*, 2007; Poot *et al.,* 2012). Sampling related individuals or the same individuals at a site would increase *σ*^2^, which was small for most measures (Table 3). As such, our sampling seems independent and appropriate for the species.

Cliff-dwelling endemics like *H. brandegeei* and *O. sinensis* have their own root morphologies and strategies that include investing a larger portion of their biomass in roots (Fig. 4), a relatively fast distribution of roots, and lower specific root length to allow them to efficiently exploit the edaphic conditions their roots encounter (Poot *et al.*, 2012). Although relatively unique, non-cliff-dwelling plants can employ related rooting strategies to exploit bedrock resources, even to access resources in fissures as small as 100 μm (Zwieniecki and Newton, 1995). Once established, microhabitats may buffer populations from future climate change if available moisture persists (e.g. Patsiou *et al.*, 2014). Furthermore, the rooting patterns of individual plants as well as neighbouring species require further investigation since biotic interactions have been shown to shape communities in un-related outcrop systems (Houle and Phillips, 1989; Wiser *et al.*, 1996) and alpine cushion species globally (Cavieres *et al.*, 2007; Antonsson *et al.*, 2009; Reid *et al.*, 2010). This may help to explain the observed-within maternal line variability we documented in the present study of *H. brandegeei*.

Only approximately 12% of seeds germinated in our experiment. This may be due to dormancy that has yet to be studied in *H. brandegeei* or a stratification requirement that was not sufficiently met. These low rates of viability are similar to other alpine species (Bliss, 1958; Stanton and Galen, 1997). It is likely that seed germination was at least partially hindered by dark conditions the seeds experienced under soil. Chasmophytic alpine species are typically exposed to intense light that stimulates germination in their natural setting (Shimono and Kudo, 2005; Brusa *et al.*, 2007). Nonetheless, *H. brandegeei* individuals produce large numbers of seeds that are wind dispersed. This likely determines population structure and individual plant occurrence across the landscape (Nathan and Muller-Landau, 2000). *H. brandegeei* produces only disc achenes with thin pericarps, which facilitates wind dispersal and allows for relatively rapid germination (Flint and Palmblad, 1978). This seed type and dispersal strategy likely reduces some of the negative effects of inbreeding that are expected in a range-restricted endemic like *H. brandegeei* (Gibson and Tomlinson, 2002); though no known genetic studies have been conducted on *H. brandegeei* to date.

Our study demonstrates the role the phenotypic variation can play in shaping individual and population responses of a rare, endemic alpine plant experiencing warming and drought. *H. brandegeei* reduced overall investment in building plant structures and, instead, maintained fewer tissues in responses to simulated climate change. The combined effects of warming and drought increased responses, though these were not significant for most traits measured. However, the individual effects of warming and drought stress were overall too much for the species and caused high mortality. This is most clearly owed to the already sensitive life history of *H. brandegeei* and that establishment is dependent on seeds landing in a suitable crevice or fissure across disparate rock outcroppings. This alone likely makes *H. brandegeei* populations more susceptible to human impacts like climate change (Jump and Peñuelas, 2005).

Finally, it seems imperative that rare, endemic species should be assigned high priority for research efforts. Successful conservation efforts for rare and endemic taxa are dependent upon population estimates, distribution surveys and habitat characterizations that are currently lacking for many of Baja California’s endemic species (Vanderplank *et al.*, 2018) and other sensitive plant and animal species around the globe (Kruckeberg and Rabinowitz, 1985; Harper *et al.*, 2016). Studies like ours provide a small glimpse at species responses during a particular life stage, albeit an important phase for this likely long-lived cushion species. Additional work is required to examine *H. brandegeei* in its natural environment. Research should address the role microhabitats play in germination and establishment and the subsequent abiotic and biotic interactions that occur throughout an individual plant’s life cycle. Future research should also determine current population structure and potential impacts climate change may have on gene flow across this fragmented landscape.

## Author contributions

D.W. and M.L. conceived and designed the experiments. D.W., K.C. and M.L. collected the data. D.W. and M.L. analyzed the data. D.W. and M.L. drafted the manuscript. All authors contributed to writing the final version of the manuscript.

## Supplementary Material

Winkler_et_al_Heterotheca_Supplementary_Information_coz076
