# Supplementary material for: Early life history responses and phenotypic shifts in a rare endemic plant responding to climate change
Source: Conserv Physiol. 2019 Oct 31;7(1):coz076. doi: 10.1093/conphys/coz076 (PMC6822542; doi:10.1093/conphys/coz076)
Supplement: Winkler_et_al_Heterotheca_Supplementary_Information_coz076 [file winkler_et_al_heterotheca_supplementary_information_coz076.doc]

**Supplementary Information**


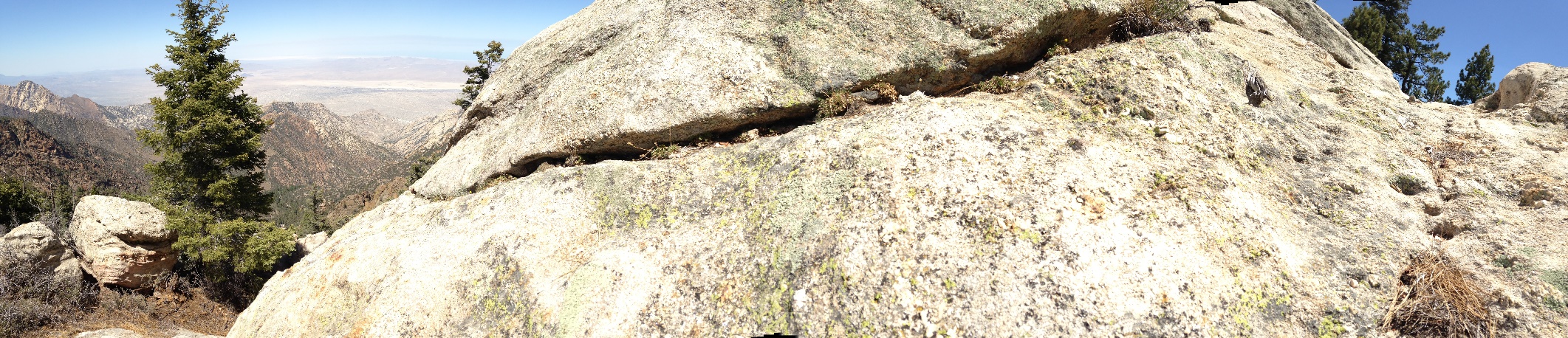


Figure S1. Rock crevice in Sierra de San Pedro Mártir National Park, Baja California, México. Plants including *Heterotheca brandegei* can be found growing inside the crevice.

Table S1. Pairwise correlation coefficients for target response variables. We retained variables that had correlation coefficients under |0.75| and selected total biomass to stand for the other biomass variables.

|  | **agb** | **bgb** | **total biomass** | **# leaves** | **% C in leaves** | **WUE** | **SLA** |
| --- | --- | --- | --- | --- | --- | --- | --- |
| **agb** | - | 0.79 | 0.86 | 0.72 | 0.45 | 0.18 | -0.07 |
| **bgb** | 0.79 | - | 0.99 | 0.69 | 0.34 | -0.08 | 0.06 |
| **total biomass** | 0.86 | 0.99 | - | 0.73 | 0.38 | -0.02 | 0.03 |
| **# leaves** | 0.72 | 0.69 | 0.73 | - | 0.37 | 0.05 | 0.23 |
| **% C in leaves** | 0.45 | 0.34 | 0.38 | 0.37 | - | -0.42 | -0.06 |
| **WUE** | 0.18 | -0.08 | -0.02 | 0.05 | -0.42 | - | -0.25 |
| **SLA** | -0.07 | 0.06 | 0.03 | 0.23 | -0.06 | -0.25 | - |

Table S2. Summary statistics for additional plant traits not included in analyses. Area measurements are reported in mm^2^, weights are in mg, specific leaf area measurements are mm^2^ mm^-1^, and root length is in mm.

| trait | ambient | n | warming | n | drought | n | w + d | n |
| --- | --- | --- | --- | --- | --- | --- | --- | --- |
| Total leaf area | 959.62 ± 143.45 | 12 | 528.57 ± 140.81 | 11 | 352.99 ± 44.86 | 11 | 526.95 ± 158.58 | 10 |
| Average leaf area | 56.96 ± 14.74 | 12 | 45.06 ± 12.57 | 11 | 35.24 ± 10.21 | 11 | 70.63 ± 17.04 | 10 |
| Maximum leaf area | 92.22 ± 14.14 | 12 | 65.61 ± 12.03 | 11 | 49.89 ± 9.15 | 11 | 84.19 ± 18.77 | 10 |
| Minimum leaf area | 3.63 ± 1.25 | 12 | 5.44 ± 2.12 | 11 | 5.33 ± 1.69 | 11 | 8.59 ± 2.36 | 10 |
| Stem dry weight | 56.26 ± 9.80 | 12 | 32.78 ± 8.59 | 11 | 28.65 ± 5.95 | 11 | 20.80 ± 4.60 | 10 |
| Total leaf dry weight | 53.58 ± 7.71 | 12 | 33.06 ± 9.84 | 11 | 18.82 ± 2.94 | 11 | 25.61 ± 7.85 | 10 |
| Average leaf dry weight | 3.69 ± 0.42 | 12 | 2.52 ± 0.33 | 11 | 23.12 ± 2.71 | 11 | 28.35 ± 7.42 | 9 |
| SLA leaf #1 | 18.75 ± 2.29 | 11 | 18.89 ± 3.25 | 11 | 23.12 ± 2.71 | 11 | 28.35 ± 7.41 | 9 |
| SLA leaf #2 | 16.78 ± 2.51 | 11 | 21.85 ± 4.44 | 11 | 19.36 ± 3.86 | 11 | 22.57 ± 3.02 | 9 |
| SLA leaf #3 | 17.25 ± 1.88 | 11 | 19.57 ± 3.54 | 11 | 19.89 ± 3.34 | 11 | 19.33 ± 2.94 | 9 |
| SLA leaf #4 | 16.72 ± 2.54 | 11 | 19.41 ± 4.15 | 11 | 15.83 ± 3.00 | 11 | 23.30 ± 5.23 | 8 |
| SLA leaf #5 | 14.36 ± 1.26 | 11 | 13.73 ± 1.54 | 10 | 22.36 ± 7.95 | 11 | 22.75 ± 4.55 | 9 |
| Maximum SLA | 21.48 ± 2.15 | 12 | 28.99 ± 5.21 | 11 | 33.10 ± 7.09 | 11 | 35.59 ± 8.19 | 8 |
| Minimum SLA | 13.14 ± 1.52 | 12 | 12.06 ± 1.44 | 11 | 10.82 ± 0.87 | 11 | 15.76 ± 2.66 | 8 |
| Root length | 239.33 ± 23.17 | 12 | 223.18 ± 32.74 | 11 | 203.45 ± 18.08 | 11 | 211.30 ± 30.58 | 10 |

Table S3. Phenological traits including the number of days to germinations and the emergence of the first five true leaves. Mean and SEM by treatment.

| **phenological trait** | 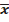 **± SEM** | ***n*** |
| --- | --- | --- |
| # days to germination | 8.85 ± 0.33 | 171 |
| 1^st^ leaf | 8.07 ± 0.23 | 161 |
| 2^nd^ leaf | 10.62 ± 0.25 | 154 |
| 3^rd^ leaf | 16.07 ± 0.36 | 137 |
| 4^th^ leaf | 20.43 ± 0.46 | 114 |
| 5^th^ leaf | 25.89 ± 0.55 | 79 |

Table S4. Variance (*σ*^2^) explaining the number of days to germination and emergence of the first true leaf. Variance components include the amount of variation explained by differences between locations, between maternal lines within locations, between individuals within maternal lines, and by residual error.

|  | **Between locations** | **Between maternal lines within locations** | **Between individuals within maternal lines** | **Residual error** |
| --- | --- | --- | --- | --- |
| # days to germination | 0.05 | 0.04 | 0.19 | 0.71 |
| 1^st^ leaf | 0 | 0.05 | 0.15 | 0.81 |

Table S5. Results of linear mixed effects models predicting total biomass with warming (w) and drought (d) treatments, their interaction, and plant age as fixed effects. Location, maternal line, and individual plant ID are included as nested random effects. AICc are Akaike Information Criterion values corrected for small sample sizes. Marginal *r^2^* estimated the predictive power of fixed effects. Conditional *r^2^* estimates the predictive power of both fixed and random effects. *w*_i_ are Akaike weights, which indicate the probability of each model being the best fit relative to others shown.

| **model** | **AICc** | **k** | **marginal *r^2^*** | **conditional *r^2^*** | **∆AICc** | ***w*_i_** |
| --- | --- | --- | --- | --- | --- | --- |
| d × w + age | 525.801 | 4 | 0.57 | 0.59 | 0.00 | 0.998 |
| d + w + age | 538.654 | 3 | 0.57 | 0.61 | 12.85 | 0.002 |
| d + w | 562.066 | 2 | 0.26 | 0.32 | 36.27 | 0 |
| d + age | 551.425 | 2 | 0.55 | 0.60 | 25.63 | 0 |
| w + age | 560.931 | 2 | 0.41 | 0.41 | 35.13 | 0 |
| d | 575.255 | 1 | 0.21 | 0.27 | 49.45 | 0 |
| w | 582.774 | 1 | 0.05 | 0.17 | 56.97 | 0 |
| age | 573.250 | 1 | 0.38 | 0.38 | 47.45 | 0 |
| intercept only | 595.441 | 0 | 0.00 | 0.19 | 69.64 | 0 |

Table S6. Results of linear mixed effects models predicting the total number of leaves produced with warming (w) and drought (d) treatments, their interaction, and plant age as fixed effects. Location, maternal line, and individual plant ID are included as nested random effects. AICc are Akaike Information Criterion values corrected for small sample sizes. Marginal *r^2^* estimated the predictive power of fixed effects. Conditional *r^2^* estimates the predictive power of both fixed and random effects. *w*_i_ are Akaike weights, which indicate the probability of each model being the best fit relative to others shown.

| **model** | **AICc** | **k** | **marginal *r^2^*** | **conditional *r^2^*** | **∆AICc** | ***w*_i_** |
| --- | --- | --- | --- | --- | --- | --- |
| d × w + age | 97.66 | 4 | 0.43 | 0.62 | 0.00 | 0.43 |
| d + w + age | 99.35 | 3 | 0.42 | 0.58 | 1.69 | 0.185 |
| d + w | 98.08 | 2 | 0.30 | 0.60 | 0.42 | 0.348 |
| d + age | 104.96 | 2 | 0.32 | 0.57 | 7.30 | 0.011 |
| w + age | 107.07 | 2 | 0.29 | 0.43 | 9.41 | 0.004 |
| d | 104.37 | 1 | 0.17 | 0.54 | 6.72 | 0.015 |
| w | 106.28 | 1 | 0.14 | 0.40 | 8.63 | 0.006 |
| age | 111.64 | 1 | 0.18 | 0.42 | 13.98 | 0 |
| intercept only | 111.29 | 0 | 0.00 | 0.38 | 13.63 | 0 |

Table S7. Results of linear mixed effects models predicting the average specific leaf area of the five largest leaves with warming (w) and drought (d) treatments, their interaction, and plant age as fixed effects. Location, maternal line, and individual plant ID are included as nested random effects. AICc are Akaike Information Criterion values corrected for small sample sizes. Marginal *r^2^* estimated the predictive power of fixed effects. Conditional *r^2^* estimates the predictive power of both fixed and random effects. *w*_i_ are Akaike weights, which indicate the probability of each model being the best fit relative to others shown.

| **model** | **AICc** | **k** | **marginal *r^2^*** | **conditional *r^2^*** | **∆AICc** | ***w*_i_** |
| --- | --- | --- | --- | --- | --- | --- |
| d × w + age | 276.23 | 4 | 0.48 | 0.48 | 0.00 | 0.896 |
| d + w + age | 281.00 | 3 | 0.49 | 0.49 | 4.78 | 0.082 |
| d + w | 301.80 | 2 | 0.05 | 0.05 | 25.57 | 0 |
| d + age | 284.60 | 2 | 0.49 | 0.49 | 8.38 | 0.014 |
| w + age | 285.55 | 2 | 0.48 | 0.48 | 9.33 | 0.008 |
| d | 306.33 | 1 | 0.04 | 0.04 | 30.10 | 0 |
| w | 307.48 | 1 | 0.01 | 0.01 | 31.25 | 0 |
| age | 294.62 | 1 | 0.03 | 0.80 | 18.39 | 0 |
| intercept only | 311.90 | 0 | 0.00 | 0.00 | 35.67 | 0 |

Table S8. Results of linear mixed effects models predicting the % carbon in leaf tissues with warming (w) and drought (d) treatments, their interaction, and plant age as fixed effects. Location, maternal line, and individual plant ID are included as nested random effects. AICc are Akaike Information Criterion values corrected for small sample sizes. Marginal *r^2^* estimated the predictive power of fixed effects. Conditional *r^2^* estimates the predictive power of both fixed and random effects. *w*_i_ are Akaike weights, which indicate the probability of each model being the best fit relative to others shown.

| **model** | **AICc** | **k** | **marginal *r^2^*** | **conditional *r^2^*** | **∆AICc** | ***w*_i_** |
| --- | --- | --- | --- | --- | --- | --- |
| d × w + age | 251.25 | 4 | 0.23 | 0.95 | 0.00 | 0.998 |
| d + w + age | 263.69 | 3 | 0.20 | 1.00 | 12.44 | 0.002 |
| d + w | 270.12 | 2 | 0.17 | 1.00 | 18.87 | 0 |
| d + age | 277.46 | 2 | 0.16 | 1.00 | 26.21 | 0 |
| w + age | 281.14 | 2 | 0.10 | 0.81 | 29.89 | 0 |
| d | 283.54 | 1 | 0.11 | 1.00 | 32.29 | 0 |
| w | 286.69 | 1 | 0.06 | 0.81 | 35.44 | 0 |
| age | 294.62 | 1 | 0.03 | 0.80 | 43.37 | 0 |
| intercept only | 299.77 | 0 | 0.00 | 0.79 | 48.52 | 0 |

Table S9. Results of linear mixed effects models predicting leaf-level water-use efficiency with warming (w) and drought (d) treatments, their interaction, and plant age as fixed effects. Location, maternal line, and individual plant ID are included as nested random effects. AICc are Akaike Information Criterion values corrected for small sample sizes. Marginal *r^2^* estimated the predictive power of fixed effects. Conditional *r^2^* estimates the predictive power of both fixed and random effects. *w*_i_ are Akaike weights, which indicate the probability of each model being the best fit relative to others shown.

| **model** | **AICc** | **k** | **marginal *r^2^*** | **conditional *r^2^*** | **∆AICc** | ***w*_i_** |
| --- | --- | --- | --- | --- | --- | --- |
| d × w + age | 92.45 | 4 | 0.33 | 0.97 | 0.00 | 0.999 |
| d + w + age | 108.06 | 3 | 0.14 | 0.89 | 15.61 | 0 |
| d + w | 109.10 | 2 | 0.03 | 0.61 | 16.65 | 0 |
| d + age | 109.82 | 2 | 0.14 | 0.90 | 17.38 | 0 |
| w + age | 110.31 | 2 | 0.14 | 0.87 | 17.87 | 0 |
| d | 112.17 | 1 | 0.01 | 0.66 | 19.72 | 0 |
| w | 111.72 | 1 | 0.01 | 0.65 | 19.28 | 0 |
| age | 112.32 | 1 | 0.12 | 0.88 | 19.87 | 0 |
| intercept only | 114.54 | 0 | 0.00 | 0.71 | 22.10 | 0 |

Table S10. Results of logistic mixed effects models predicting mortality with warming (w) and drought (d) treatments, their interaction, and plant age as fixed effects. Location, maternal line, and individual plant ID are included as nested random effects. AICc are Akaike Information Criterion values corrected for small sample sizes. Marginal *r^2^* estimated the predictive power of fixed effects. Conditional *r^2^* estimates the predictive power of both fixed and random effects. *w*_i_ are Akaike weights, which indicate the probability of each model being the best fit relative to others shown.

| **model** | **AICc** | **k** | **∆AICc** | ***w*_i_** |
| --- | --- | --- | --- | --- |
| d × w | 148.58 | 3 | 1.23 | 0.288 |
| d + w | 147.35 | 2 | 0.00 | 0.533 |
| d | 150.57 | 1 | 3.23 | 0.106 |
| w | 151.56 | 1 | 4.22 | 0.065 |
| intercept only | 155.60 | 0 | 8.26 | 0.009 |
